# Novel combination of mitochondrial division inhibitor 1 (mdivi-1) and platinum agents produces synergistic pro-apoptotic effect in drug resistant tumor cells

**DOI:** 10.18632/oncotarget.1944

**Published:** 2014-05-04

**Authors:** Wei Qian, Jingnan Wang, Vera Roginskaya, Lee A. McDermott, Robert P. Edwards, Donna B. Stolz, Fabien Llambi, Douglas R. Green, Bennett Van Houten

**Affiliations:** ^1^ Department of Pharmacology & Chemical Biology, School of Medicine, University of Pittsburgh and Hillman Cancer Center, University of Pittsburgh Cancer Institute, Pittsburgh, PA 15213, USA; ^2^ Tsinghua University School of Medicine, Tsinghua University, Haidian District, Beijing 100084, China; ^3^ Department of Pharmaceutical Sciences, School of Pharmacy, University of Pittsburgh and University of Pittsburgh Drug Discovery Institute, Pittsburgh, PA 15213, USA; ^4^ Department of Obstetrics & Gynecology, University of Pittsburgh Medical Center, Pittsburgh, PA 15213, USA; ^5^ Department of Cell Biology, University of Pittsburgh, Pittsburgh, PA 15261, USA; ^6^ Department of Immunology, St. Jude Children's Research Hospital, Memphis, TN 38105, USA

**Keywords:** Platinum resistance, mdivi-1, replication stress, Noxa, mitochondrial swelling

## Abstract

Overcoming platinum drug resistance represents a major clinical challenge in cancer treatment. We discovered a novel drug combination using cisplatin and a class of thioquinazolinone derivatives including mdivi-1 (mitochondrial division inhibitor-1), that induces synergistic apoptosis in platinum resistant tumor cells, including those from cisplatin-refractory endstage ovarian cancer patients. However, through study of the combination effect on Drp1 (the reported target of mdivi-1) knockout MEF cells and the functional analysis of mdivi-1 analogs, we revealed that the synergism between mdivi-1 and cisplatin is Drp1-independent. Mdivi-1 impairs DNA replication and its combination with cisplatin induces a synergistic increase of replication stress and DNA damage, causing a preferential upregulation of a BH3-only protein Noxa. Mdivi-1 also represses mitochondrial respiration independent of Drp1, and the combination of mdivi-1 and cisplatin triggers substantial mitochondrial uncoupling and swelling. Upregulation of Noxa and simultaneous mitochondrial swelling causes synergistic induction of mitochondrial outer membrane permeabilization (MOMP), proceeding robust mitochondrial apoptotic signaling independent of Bax/Bak. Thus, the novel mode of MOMP induction by the combination through the “dual-targeting” potential of mdivi-1 on DNA replication and mitochondrial respiration suggests a novel class of compounds for platinum-based combination option in the treatment of platinum as well as multidrug resistant tumors.

## INTRODUCTION

The platinum-based anticancer drugs, including cisplatin and carboplatin, are currently among the most potent and widely used chemotherapeutic agents. They are used for treating a variety of cancers, including testicular, ovarian, colorectal, bladder, lung, and head and neck cancers [1]. The major limitations for the clinical application of these platinum drugs are their inherent toxicities, as well as, the high incidence of intrinsic and acquired drug resistance by tumors [2, 3]. Development of cisplatin resistance is often associated with multidrug resistant phenotype. In particular for ovarian cancer, which is the leading cause of death from gynecologic malignancies, platinum compounds-based therapies are the current global standard [4]. The initial treatment response rate to cisplatin in ovarian cancer patients can be up to 70% [5]. Unfortunately, 70% of those patients who responded to cisplatin experience disease recurrence and eventually develop resistance to therapy, resulting in incurable disease [6]. Platinum resistance is the single most important factor after stage in determining prognosis.

The anticancer activity of cisplatin appears to rely on multiple mechanisms. The uptake of cisplatin by cells is believed to occur by both passive diffusion and a transporter-mediated process such as through copper transporter 1 (CTR1) [7]. Once inside the cell cisplatin undergoes a series of aquation reactions, in which one or both its cis-chloro ligands are replaced by water molecules due to the relatively low concentration of intracellular chloride ions, leading to the generation of positively charged highly reactive aquated cisplatin [8]. Aquated cisplatin is prone to interact with a number of intracellular macromolecules, and the most prominent mechanism underlying cisplatin-induced cell death has been demonstrated to be through formation of cisplatin-DNA adducts. The platinum atom binds to the N^7^ position of adjacent purines, primarily guanine to form 1, 2 intrastrand cross-links (PtGpGs), leading to the generation of DNA inter- and intra-strand adducts as well as DNA-protein complexes [8]. Cisplatin-induced intra-strand adducts are recognized and removed by nucleotide excision repair (NER) [9]. Cisplatin-induced DNA damage activates ATR (ataxia telangiectasia mutated (ATM)- and RAD3-related protein), leading to cell cycle arrest in the G2 phase [1]. When DNA damage is extensive and persistent, cells may undergo mitochondria-mediated apoptotic cell death [2]. The molecular mechanisms of platinum drug resistance have not been fully elucidated. It is generally considered that the resistance has multiple mechanisms depending on cell types and commonly more than one resistance mechanism is involved [1]. Cisplatin resistance can be the result of alterations in any of the steps required for cisplatin action, and has been attributed to reduced cellular accumulation of cisplatin, enhanced repair activities against cisplatin-DNA adducts, increased tolerance to cisplatin-induced DNA damage, and failure of apoptotic pathway.

Small molecule inhibitors such as ATR and PARP inhibitors, which prevent repair of cisplatin-induced DNA lesions, when combined with cisplatin have shown promise both preclinically and clinically [10, 11]. As chemosensitizers, such small molecules provide vital therapeutic approach in managing certain types of tumors. We have shown previously that mdivi-1, an inhibitor of mitochondrial division protein Drp1, induces gross genome instability in tumor cells [12]. Mdivi-1 has been reported to block the self-assembly of Drp1 and retard apoptosis by preventing Bax/Bak-dependent mitochondrial outer membrane permeabilization (MOMP) [13]. Due to its safety and protective benefits that have been shown in vitro and in vivo [14-17], mdivi-1 represents a novel class of therapeutics for stroke, myocardial infarction and neurodegenerative diseases [13].

In this study, we present a novel finding that the combination of cisplatin and mdivi-1 possesses unusual anticancer potency by acting synergistically in inducing robust apoptosis in cisplatin and multidrug resistant tumor cells, in a Drp1-independent manner. We identified that mdivi-1 directly causes replication stress and mitochondrial dysfunction. In combination with cisplatin, these effects were greatly enhanced leading to synergistic induction of MOMP independent of Bax and Bak. Since loss of Bax and Bak causes complete resistance to cisplatin [18], the ability of our combination strategy in inducing MOMP in a Bax/Bak-independent manner appears to be a crucial mechanism in overcoming cisplatin resistance.

## RESULTS

### Combination of cisplatin and mdivi-1 produces a synergistic pro-apoptotic effect in tumor cells that have inherent or acquired resistance to cisplatin

**Figure 1:**
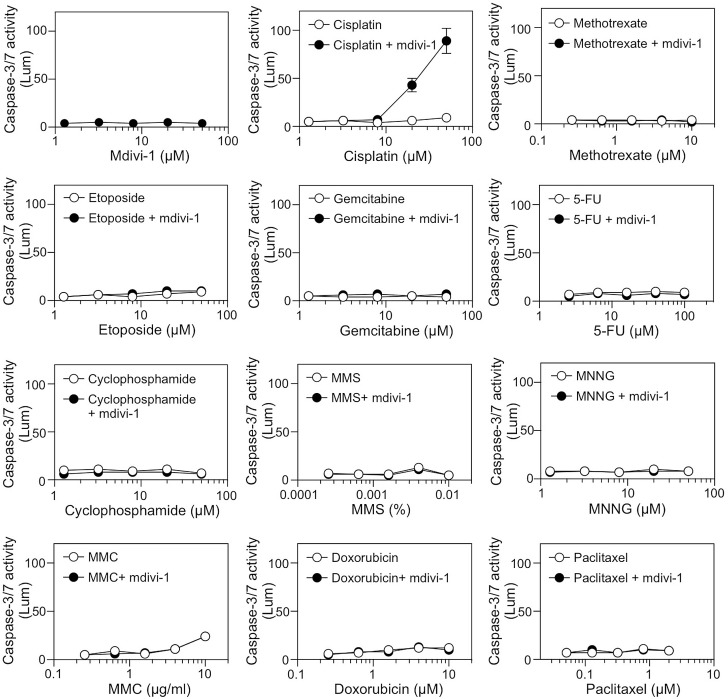
Identification of cisplatin as an agent whose efficacy is specifically enhanced by mdivi-1 Multidrug-resistant MDA-MB-231 breast carcinoma cells were treated with increasing doses of mdivi-1 alone, a series of anticancer drugs alone, or the combination of increasing doses of anticancer drugs with 20 μM mdivi-1 for 20 h Apoptosis was determined by measuring the activity of caspase-3/7. The drugs tested were platinum agent, cisplatin; antifolate agent, methotrexate; topoisomerase II inhibitor, etoposide; cytidine analog, gemcitabine; pyrimidine analog, 5-FU; alkylating agents, cyclophosphamide, MMS and MNNG; DNA crosslinking agent, MMC; DNA intercalating agent, doxorubicin; and antimitotic drug, paclitaxel. These data represent the mean ± s.d.; n = 4.

We have shown previously that mdivi-1 induces genome instability in several types of cancer cells including MDA-MB-231 breast carcinoma cells [12]. MDA-MB-231 cells are hormone receptor- and ERBB2-negative “triple negative” and multidrug resistant [19]. Currently no tailored therapy exists for such type of cancer [11]. We therefore used MDA-MB-231 cells as a model to identify chemotherapeutic agents that when combined with mdivi-1 are able to reverse the drug resistance in these cells. By using a caspase-3/7 activity assay, we tested the effect of combining mdivi-1 with a series of clinically highly significant drugs with disparate actions, and found that cisplatin is a unique agent whose effect can be greatly enhanced by mdivi-1 (Fig. 1).

**Figure 2:**
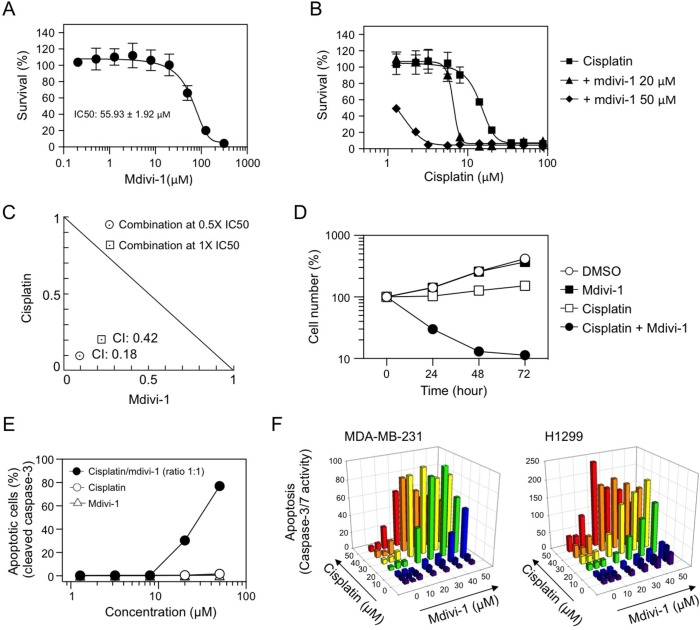
Combination of cisplatin and mdivi-1 produces synergistic pro-apoptotic effect in multidrug resistant tumor cells (A) MDA-MB-231 cells were treated with increasing doses of mdivi-1 for 72 h The survival was assessed by MTS assay. These data represent the mean ± s.d.; n = 4. (B) MDA-MB-231 cells were treated with increasing doses of cisplatin alone or the combination with 20 μM or 50 μM of mdivi-1 for 72 h. The survival was assessed by MTS assay. (C) MDA-MB-231 cells were exposed to cisplatin and mdivi-1 alone or combination at their IC50 or 0.5 fold of their IC50. The combination index (CI) and the normalized isobologram were generated using CompuSyn. (D) MDA-MB-231 cells were treated with DMSO, 50 μM cisplatin alone, 50 μM mdivi-1 alone, or the combination for 2 h. The compounds were then washed out and the cell number was determined every 24 h by CyQuant assay. (E) MDA-MB-231 cells were treated with agents alone, or the combination at a ratio of 1:1 for 20 h. The number of apoptotic cells was determined with antibody recognizing cleaved caspase-3 followed by flow cytometry. These data represent the mean ± s.d.; n = 3. (F) MDA-MB-231 and H1299 cells were treated with various combinations of cisplatin and mdivi-1 at indicated concentrations. After 20 h, apoptosis was determined by the activity of caspase-3/7.

In order to understand the interactions between cisplatin and mdivi-1, we performed a thorough characterization of their combination effect. Mdivi-1 alone inhibited the growth of MDA-MB-231 cells with an IC50 of 55.93 ± 1.92 μM (Fig. 2A). The IC50 of cisplatin for MDA-MB-231 cells is 14.29 ± 1.03 μM (Fig. 2B). In the presence of mdivi-1, the survival curve was shifted towards the left dose-dependently compared to cisplatin alone treatment (Fig. 2B), indicating that mdivi-1 is able to enhance the efficacy of cisplatin and reduce the doses of effective cisplatin into low micromolar range. The nature of the interactions between cisplatin and mdivi-1 was further evaluated based on the median-effect principle of Chou and Talalay [20]. The value of the combination index (CI), which is 0.42 for the drugs combined at their IC50 and 0.18 at 0.5 fold of their IC50 (Fig. 2C), indicated that the combination produces a synergistic anti-proliferative effect. Furthermore, a short-term two-hour exposure of cells to the combination of cisplatin and mdivi-1 is sufficient to dramatically decrease cell number within 24 h (70% reduction compared to cisplatin alone treatment) (Fig. 2D), indicating rapid induction of cell death. This is in sharp contrast to the two-hour exposure of cells to cisplatin alone, which only caused a slight delay in cell growth. Cleavage of caspase-3 was then used to quantify apoptotic cell death. A 20 h treatment with the combination of cisplatin and mdivi-1 at 1:1 ratio at 20 μM or higher triggered a dramatic increase in the number of apoptotic cells (Fig. 2E). It is also noteworthy that the synergism we observed in MDA-MB-231 cells is highly significant compared to certain known platinum-based combinations, as those cells are also resistant to the combination of cisplatin with promising molecular targeted strategies such as inhibition of ATR and PARP (Supplemental Fig. 1). The synergistic effect was also demonstrated by a dose matrix response assay in MDA-MB-231 cells and other types of cancer cells with inherent cisplatin resistance, such as non-small cell lung carcinoma H1299 cells (Fig. 2F), glioblastoma, head and neck, and melanoma cells (Supplemental Fig. 2). Furthermore, another platinum analog carboplatin was also found to have similar combination effect with mdivi-1 (Supplemental Fig. 3). We also found that the combination effect is not dependent on p53 status of the cells (Supplemental Fig. 4).

**Figure 3:**
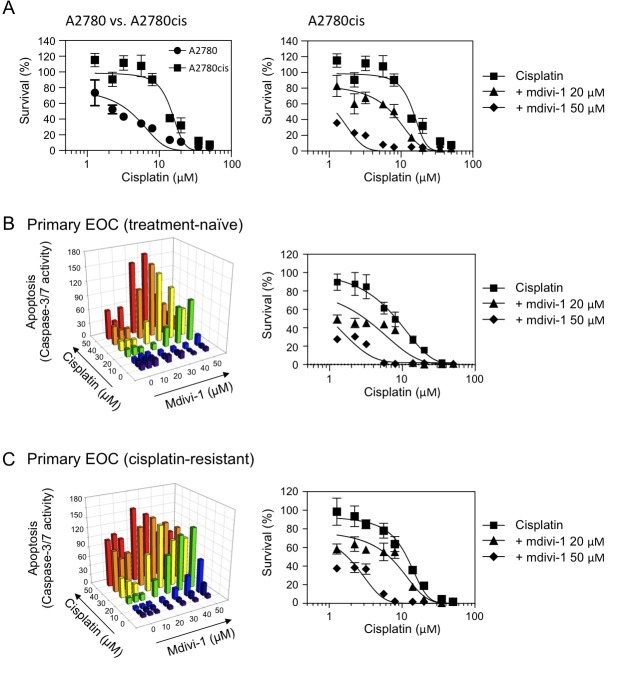
The combination of cisplatin and mdivi-1 efficiently overcomes acquired cisplatin resistance in human ovarian cancer cells, including those from endstage cisplatin- and treatment-refractory patient (A) The comparison of cisplatin sensitivity in ovarian cancer cells A2780 and their derivative cisplatin-resistant A2780cis cells (left panel) A2780cis cells were treated with cisplatin alone or in combination with mdivi-1 for 72 h (right panel). The survival was determined by MTS assay. (B and C) Ex vivo drug sensitivity assay using primary EOC cells isolated from ascites fluid of ovarian cancer patients (B, treatment-naïve; C, cisplatin-resistant). Apoptosis was measured by caspase-3/7 activity assay after 20 h exposure and the survival was measured by MTS assay after 72 h exposure.

Since acquired cisplatin resistance is also a major obstacle to successful platinum-based therapy, we next tested the effect of the combination on tumor cells with acquired cisplatin resistance. The A2780cis ovarian cancer cell line (left panel of Fig. 3A) is a cisplatin-resistant A2780 derivative, established by chronic exposure of the parental cisplatin-sensitive A2780 cell line (established from ovarian epithelial tumor tissue from an untreated patient) to increasing concentrations of cisplatin. A2780cis is also cross-resistant to melphalan, adriamycin and irradiation. Similarly as we observed in Fig. 2B, mdivi-1 greatly increased cisplatin efficacy in A2780cis cells (right panel of Fig. 3A). Ovarian cancer patients with ascites are more frequently platinum resistant than patients without ascites [21]. The ex vivo drug sensitivity assay using tumor cells isolated from patient ascites is capable of predicting outcomes of patients and assisting oncologists in making treatment decision selecting assay-sensitive agents [22]. We thus tested the effect of the combination of cisplatin and mdivi-1 on primary epithelial ovarian cancer (EOC) cells derived from patient ascites fluids. In Fig. 3B, primary EOC cells were isolated from the ascites of a patient who had not been treated with cisplatin but showed relative resistance to cisplatin (IC50 ≈ 7.97 μM). In Fig. 3C, primary EOS cells were isolated from a patient who had developed clinically-defined cisplatin resistance due to previous treatments (IC50 ≈ 11.64 μM). Both the caspase-3/7 activity assay and MTS assay revealed that the combination with mdivi-1 dramatically enhanced cisplatin efficacy (Fig. 3B and C). It is also noteworthy that the doses of cisplatin we tested in our ex vivo analysis are clinically relevant, since they are in the range achievable by intraperitoneal cisplatin administration [23, 24]. Intraperitoneal administration has been shown to increase patient survival compared with intravenous administration, as peritoneal cavity is the main site of disease in ovarian cancer [24].

### Mdivi-1 enhances cisplatin sensitivity through Drp1-independent mechanisms

**Figure 4:**
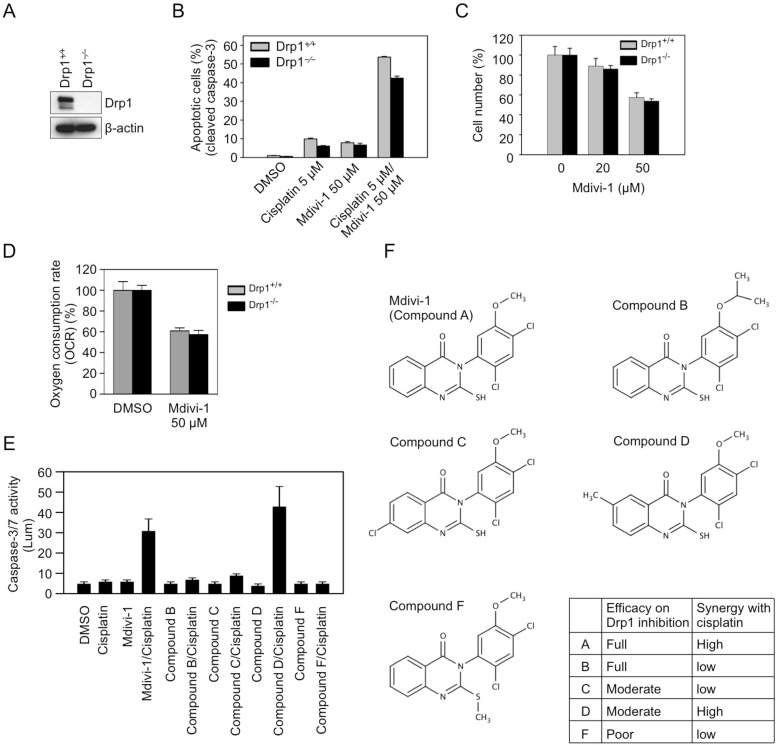
Mdivi-1 enhances cisplatin sensitivity through Drp1-independent mechanisms (A) Confirmation of the depletion of Drp1 in Drp1 knockout SV40-transformed MEF cells by western blot (B) Drp1 wild-type (Drp1 +/+) and knockout (Drp1 −/−) MEF cells were treated as indicated for 20 h, and the number of apoptotic cells was determined by antibody recognizing cleaved caspase-3. These data represent the mean ± s.d.; n = 3. (C) Drp1 wild-type and knockout MEF cells were treated with mdivi-1 for 24 h, and the cell number was determined by CyQuant assay. (D) OCR was measured after 1 h treatment of Drp1 wild-type and knockout MEF cells with DMSO or mdivi-1. (E) The combination effect of cisplatin with four analogs of mdivi-1. MDA-MB-231 cells were treated with 20 μM of agents alone or in combination for 20 h. (F) The structures of mdivi-1 and its analogs tested in panel E.

To investigate whether the synergistic effect of the combination of cisplatin and mdivi-1 is dependent on the inhibition of Drp1 by mdivi-1, we evaluated the combination effect on Drp1 wild-type (WT) and knockout (KO) SV40-transformed MEF cells (Fig. 4A). Surprisingly, after treatment with the combination, both Drp1 WT and KO cells showed similar increase in apoptosis (Fig. 4B), indicating that the synergism is Drp1-independent. We also identified other Drp1-independent effects of mdivi-1, including inhibition of cell proliferation (Fig. 4C) and mitochondrial respiration (Fig. 4D). We further evaluated the combination effect of cisplatin with a series of mdivi-1 analogs that have been reported to have differential potency in inhibiting Drp1 (Fig. 4E and F). Compound B was reported to have full efficacy as mdivi-1 (compound A) in inhibiting Drp1 [13], but our caspase-3/7 activity assay showed that compound B only had limited effect when combined with cisplatin in enhancing apoptosis, indicating that while the i-Propyl group does not affect the efficacy on Drp1 inhibition, this group diminishes the potential for the synergism with cisplatin. Compounds C and D were described both as having moderate efficacy in inhibiting Drp1. However, we found that only compound D when combined with cisplatin had similar activity as compared to mdivi-1. Blocking SH group with methyl in compound F abolished the synergism with cisplatin, indicating the SH group is necessary for both inhibiting Drp1 and the synergism with cisplatin. Thus, the structure activity relationship (SAR) of mdivi-1 and analogs with respect to the synergism with cisplatin is not consistent with the SAR for inhibiting Drp1. Taken together, these data indicate that midiv-1 enhances cisplatin efficacy through Drp1-independent mechanisms.

### Mdivi-1 inhibits DNA replication and its combination with cisplatin enhances replication stress leading to efficient G2 phase arrest of the cell cycle

**Figure 5:**
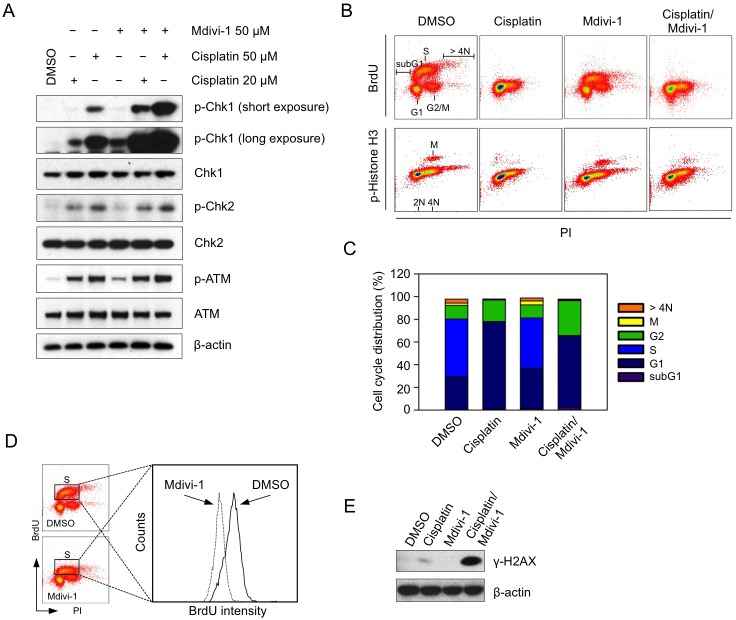
Mdivi-1 inhibits DNA replication and its combination with cisplatin enhances replication stress leading to efficient G2 phase arrest of the cell cycle (A) MDA-MB-231 cells were treated with cisplatin alone, mdivi-1 alone or the combination at the indicated concentrations for 4 h Western blot was then performed to detect the phosphorylation status of key proteins involved in DNA damage response. (B) MDA-MB-231 cells were treated with 20 μM cisplatin alone, 20 μM mdivi-1 alone or the combination for 20 h. The distribution of cell cycle was determined by triple staining of the cells with anti-BrdU, anti-phospho-Histone H3 and PI. (C) Quantification of the distribution of cell cycle analyzed in panel B. (D) The effect of mdivi-1 on the rate of DNA synthesis was analyzed by a histogram, which was generated from the data obtained in panel B, showing the changes of BrdU intensity. (E) Cells were treated as described in B and γ-H2AX was analyzed by western blot. These data represent three independent experiments.

We then sought to understand the mechanism underlying the synergistic effect of the combination. Since one of the crucial mechanisms of cisplatin-induced cell death is through replication stress-related DNA damage, we examined the DNA damage response following combination treatment in MDA-MB-231 cells. Chk1, Chk2, and ATM were phosphorylated in response to cisplatin alone (Fig. 5A). However, the combination with mdivi-1 preferentially induced dose-dependent increased phosphorylation of Chk1 (Fig. 5A), suggesting that the enhanced replication stress is an early event upon combination treatment. Notably, mdivi-1 alone at 50 μM also induced activation of Chk1 that is similar to the effect of 20 μM cisplatin alone. This indicates that mdivi-1 alone interferes with replication, and its combination with cisplatin triggers a synergistic induction of replication stress. We then analyzed the effect of the combination on cell cycle. After 20 h treatment, cisplatin alone abolished the uptake of BrdU (Fig. 5B). But rather than a G2 arrest as reported previously [25], the majority of cells accumulated in the next round of G1 phase. In contrast, the combination of cisplatin and mdivi-1 resulted in an increase of G2 phase cells (30.75% compared to 18.97% in cells treated with cisplatin alone). Despite a slight decrease in the number of S phase cells following mdivi-1 treatment (44.23% compared to 50.84% in DMSO-treated cells) (Fig. 5C), the amount of BrdU that was incorporated into DNA (BrdU intensity) was substantially decreased (Fig. 5D), further indicating that mdivi-1 has direct and profound impact on DNA replication. Thus, the enhanced replication stress by the combination during S phase caused a more efficient G2 arrest. DNA strand breaks are also enhanced, as shown by the high level of γ-H2AX in the combination-treated cells (Fig. 5E).

### The combination of cisplatin and mdivi-1 preferentially upregulates Noxa, and through promoting mitochondrial dysfunction and swelling enhances MOMP and mitochondrial apoptotic signaling in a Bax/Bak-independent manner

**Figure 6:**
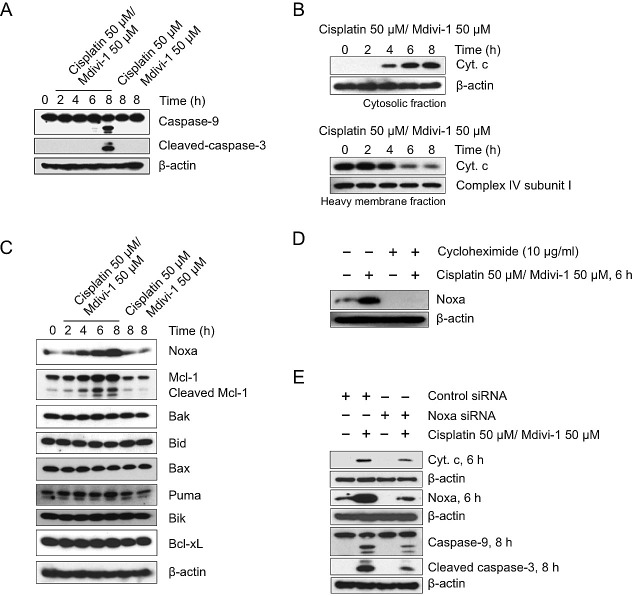
The combination of cisplatin and mdivi-1 preferentially upregulates Noxa and enhances subsequent mitochondrial apoptotic signaling (A) H1299 cells were treated with cisplatin alone, mdivi-1 alone, or the combination of cisplatin and mdivi-1 as indicated Western blot was then performed to detect the cleavage of caspase-9 and -3. (B) Cytochrome c release from mitochondria into cytosol. H1299 cells were treated with the combination of cisplatin and mdivi-1 at 50 μM with the presence of 20 μM caspase inhibitor Q-VD-OPH for the indicated time. The cytosol and heavy membrane fraction were then isolated using digitonin permeabilization followed by centrifugation. The amount of cytochrome c present in each fraction was detected by western blot. (C) The changes in the levels of pro-apoptotic and anti-apoptotic Bcl-2 family proteins. (D) The effect of cycloheximide on the levels of Noxa following the combination treatment. (E) H1299 cells were transfected with control or Noxa-specific siRNA for four days, and then treated with cisplatin and mdivi-1 as indicated. Noxa knockdown efficiency, mitochondrial release of cytochrome c, and the cleavage of caspase-9 and -3 were determined by western blot. These data represent three independent experiments.

Activation of mitochondria-initiated intrinsic apoptotic signaling by cisplatin-induced DNA damage is another critical mechanism of cisplatin action [2]. We thus focused on the role of mitochondrial pathway in the combination-induced apoptosis. Caspase-9 was cleaved only upon the combination treatment (Fig. 6A). There was also a time-dependent release of cytochrome c from mitochondria (Fig. 6B), indicating the occurrence of MOMP. We then analyzed the expression of several anti-apoptotic as well as pro-apoptotic Bcl-2 family proteins. We found that the pro-apoptotic BH3-only protein Noxa was specifically and highly induced upon combination treatment (Fig. 6C), which was accompanied with the cleavage of its binding partner Mcl-1 (Fig. 6C). Cycloheximide treatment revealed that the increased level of Noxa is not due to post-translational regulation (Fig. 6D). Knockdown of Noxa reduced the release of cytochrome c and the cleavage of caspase-9 and -3 (Fig. 6E). However, the relative high knockdown efficiency of Noxa, in conjunction with the relative small reduction in cytochrome c release and caspase cleavage, suggested the activation of additional pathways that trigger MOMP.

**Figure 7:**
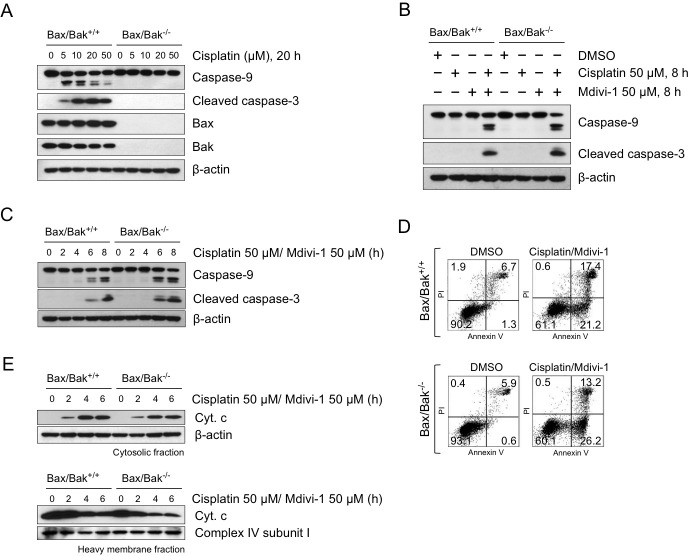
The combination of cisplatin and mdivi-1 enhances MOMP bypassing Bax/Bak-dependent mechanism (A) Bax/Bak wild-type (Bax/Bak +/+) and double knockout (Bax/Bak −/−) SV40-transformed MEF cells were treated with cisplatin at indicated concentrations for 20 h The cleavage of caspase-9 and -3 were detected by western blot. (B, C) Cleavage of caspase-9 and -3 in Bax/Bak wild-type and double knockout MEF cells after combination treatment. (D) Quantification of apoptotic cells by Annexin V and PI after 8 h treatment of combination at 50 μM. (E) Release of cytochrome c from Bax/Bak wild-type and double knockout MEF cells after combination treatment. These data represent three independent experiments.

Since Noxa promotes MOMP through Bax or Bak-mediated mechanisms [26], we next investigated the role of Bax and Bak in the induction of MOMP following the combination treatment. It is interesting to note that mdivi-1 has been shown to prevent MOMP through a Bax/Bak-dependent pathway by inhibiting Drp1 [13]. Loss of both Bax and Bak was known to render cells completely resistant to cisplatin-induced apoptosis [18]. In order to understand the role of Bax and Bak in the activation of MOMP induced by the combination, we employed Bax/Bak double knockout (DKO) SV40-transformed MEF cells. In agreement with the previous report [18], loss of both Bax and Bak rendered MEF cells resistant to cisplatin (Fig. 7A), as evidenced by the absence of cleavage of caspase-9 and -3 following 20 h treatment of cisplatin alone in Bax/Bak DKO cells. However unexpectedly, the combination of cisplatin and mdivi-1 induced the cleavage of caspase-9 and -3 in both Bax/Bak WT and DKO cells within 8 h (Fig. 7B and). Furthermore, similar number of Annexin V-positive apoptotic cells (Fig. 7D) and the release of cytochrome c (Fig. 7E) were observed in both Bax/Bak WT and DKO cells following the combination treatment. These results indicated that the combination enhances MOMP bypassing Bax/Bak-dependent mechanisms.

**Figure 8:**
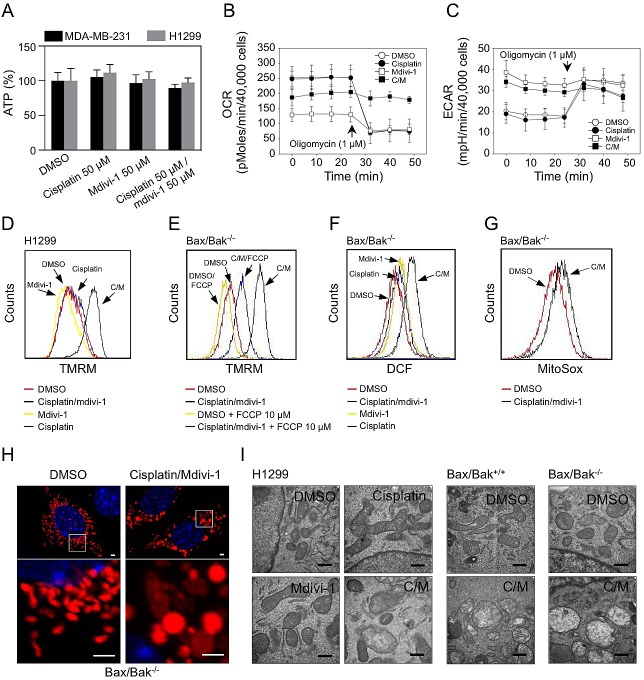
Mdivi-1 causes mitochondrial dysfunction and its combination with cisplatin induces mitochondrial swelling that triggers Bax/Bak-independent MOMP (A) MDA-MB-231 cells and H1299 cells were treated as indicated for 4 h and subjected to ATP determination These data represent the mean ± s.d.; n = 4. (B, C) OCR and ECAR were measured after 4 h treatment of H1299 cells with agents alone or the combination (C/M). (D) H1299 cells were treated as indicated for 4 h. Mitochondrial membrane potential was determined with TMRM. (E) Bax/Bak double knockout MEF cells were treated with DMSO or the combination, with or without the presence of 10 μM FCCP for 2 h. Mitochondrial membrane potential was measured as in D. (F) Bax/Bak double knockout MEF cells were treated as indicated for 2 h, ROS generation was determined with DCF-DA. (G) Bax/Bak double knockout MEF cells were pre-incubated with 5 μM MitoSox for 30 min, and then treated as indicated for 2 h followed by flow cytometry. (H) Bax/Bak double knockout MEF cells were transfected with pDsRed2-Mito plasmids. Three days after transfection, cells were treated as indicated for 4 h. Mitochondrial morphology was analyzed by confocal microscopy. The bars indicate 2 μm. (I) Cells were treated as indicated for 4h. Mitochondrial ultrastructure was analyzed by electron microscopy. The bars indicate 500 nm. In Fig. 8, the concentrations of cisplatin and mdivi-1 were used at 50 μM.

In order to understand the nature of the Bax/Bak-independent MOMP triggered by the combination, we performed a detailed analysis of mitochondrial function. No changes in intracellular ATP levels were observed in both combination and drug alone treated cells (Fig. 8A). Mdivi-1 alone suppressed mitochondrial respiration (Fig. 8B), which is consistent with the result shown in Fig. 4D. Following combination treatment, oxygen consumption rate (OCR) was decreased, but maintained at higher level than in mdivi-1 treated cells (Fig. 8B). Also intriguingly, the combination-treated cells did not respond to oligomycin, indicating significant proton leak and mitochondrial uncoupling (Fig. 8B). Both mdivi-1 and the combination treatment enhanced the extracellular acidification rate (ECAR) (Fig. 8C), further indicating mitochondrial dysfunction that caused the shift of cellular energy production from mitochondria to glycolysis. We observed a large increase in mitochondrial membrane potential only upon combination treatment (Fig. 8D), which can be prevented by an uncoupler FCCP (Fig. 8E). We also observed an enhanced production of reactive oxygen species (ROS) after combination treatment (Fig. 8F and G). Furthermore, fluorescence (Fig. 8H) and electron microscopy (Fig. 8I) revealed that the combination treatment led to increased mitochondrial size and loss of mitochondrial matrix density, indicating pronounced mitochondrial swelling, regardless of the status of Bax and Bak. These observations suggest that mitochondrial swelling and subsequent physical rupture of mitochondrial outer membrane contribute to the induction of Bax/Bak-independent MOMP in response to the combination of cisplatin and mdivi-1. Since Bax/Bak-dependent MOMP is required for the action of cisplatin alone, the ability of the combination of cisplatin and mdivi-1 in inducing MOMP in a Bax/Bak-independent manner appears to be crucial in overcoming cisplatin resistance.

## DISCUSSION

**Figure 9:**
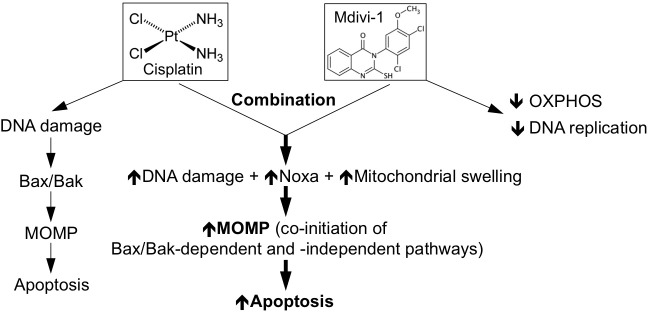
Proposed model on the mechanism underlying strong induction of apoptosis by the combination of cisplatin and mdivi-1 Cisplatin alone induces DNA damage resulting in Bax/Bak-dependent apoptosis. The combination of cisplatin and mdivi-1 is able to enhance DNA damage, upregulate Noxa expression and trigger mitochondrial swelling, which together causes co-initiation of Bax/Bak-dependent and -independent mitochondrial outer membrane permeabilization (MOMP), overcoming potential block in mitochondrial apoptotic pathway occurred in cisplatin resistant cells, thus triggering strong apoptosis.

Despite the extensive efforts that have been made to understand the complex mechanism underlying cellular resistance to platinum-based anticancer drugs, to date, overcoming this type of drug resistance by pharmacological manipulation still represents a major clinical challenge. In this study, we have discovered that mdivi-1, a thioquinazolinone compound, when combined with cisplatin is able to efficiently overcome platinum drug resistance. We also revealed novel mode of action of mdivi-1, which manifested as inhibition of DNA replication and mitochondrial respiration. The combined effect of cisplatin and mdivi-1 leads to enhanced DNA damage, upregulation of Noxa and simultaneous mitochondrial dysfunction, which result in the induction of Bax- and Bak-independent MOMP, proceeding strong activation of mitochondrial apoptotic cascade (Fig. 9). Thus, mdivi-1 and its derivative thioquinazolinone compounds represent a novel class of agents for platinum drug based combination therapies.

Mdivi-1 inhibits DNA replication leading to replication stress. The combination of cisplatin and mdivi-1 thus synergistically provokes robust replication stress-mediated signaling events, which include a more efficient G2 cell cycle arrest. G2 arrest has been shown required for engaging cell death and is thus related with cisplatin sensitivity [27, 28]. Further studies are warranted to understand the mechanism of mdivi-1 alone and its combination with cisplatin on DNA replication.

Cisplatin-DNA lesions activate DNA damage response, which eventually leads to permeabilization of the outer mitochondrial membrane via activation of the pro-apoptotic Bcl-2 family members including Bax and Bak [18]. Abrogation of mitochondrial apoptotic pathway is related with acquired multidrug resistance [29]. Noxa, a member of the pro-apoptotic BH3-only Bcl-2 protein family, can be induced by cisplatin [30], and promotes MOMP through Bax or Bak-mediated mechanisms [26]. The level of Noxa has been shown to be a central determinant of hypersensitivity to cisplatin in testicular germ cell tumors [31]. In addition, induction of Noxa is able to enhance the sensitivity of ovarian cancer cells to cisplatin [32]. Our results showed that the selective induction of Noxa is a key event for the enhanced activation of mitochondrial apoptotic signaling by the combination of cisplatin and mdivi-1. The induction of Noxa by the combination seemed to be a result of increased DNA damage response through a p53-independent mechanism, since H1299 cells are p53-null. Though mdivi-1 has been shown to block tBid-activated and Bax/Bak-mediated MOMP by inhibiting Drp1 [13], the selective induction of Noxa is able to bypass the inhibitory effect of mdivi-1 on Drp1-related MOMP, and thus contribute to the enhancement of MOMP induced by the combination of cisplatin and mdivi-1.

While Noxa plays important roles in the induction of MOMP, Noxa-mediated pathway is not a sole determinant for the combination-induced apoptosis, indicating the combination of cisplatin and mdivi-1 overcomes cisplatin resistance through targeting multiple mechanisms. We found that mdivi-1 is able to inhibit mitochondrial respiration and enhance extracellular acidification. Cisplatin is also known to inhibit the enzymatic activities of mitochondrial electron transport chain and cause depletion of mitochondrial reduced equivalents [33]. When cisplatin and mdivi-1 are combined, we observed a hyperpolarization of mitochondria, which was accompanied with mitochondrial uncoupling and generation of ROS. During apoptosis the closure of voltage-dependent anion channels (VDAC) was proposed to be related with the mitochondrial hyperpolarization, which is then followed by osmotic imbalance, physical rupture of mitochondrial outer membrane and consequent release of intermembrane space (IMS) proteins [34]. Consistent with this model, we observed extensive mitochondrial swelling and subsequent cytochrome c release independent of Bax and Bak following combination treatment.

In platinum resistant tumors, both cisplatin and mdivi-1 provoke apoptotic signaling to the extent that by each compound alone is not able to achieve efficient cell killing. In combination, however, they act in a complementary manner on the induction of MOMP by simultaneous targeting two crucial mechanisms of cisplatin action through inhibiting both DNA replication and mitochondrial function to amplify apoptotic signaling, thus producing synthetic lethal effects. This mechanism is able to bypass Bax- and Bak-mediated pathway, whose deficiencies are often seen in platinum resistant tumors. The “dual-targeting” mechanism of our combination strategy thus confers superior pro-apoptotic activity than conventional chemosensitizers that target mostly one single mechanism, such as blocking DNA repair enzymes to enhance DNA damage.

Our results elucidated pharmacologic effect of our combination strategy, thus providing a firm molecular basis for the use of mdivi-1 and its analog compounds as valuable therapeutic adjuvants. Given the organ safety and protective effect of mdivi-1 already known in small animals [35], our present work should expedite the repurposing, exploration, and development of mdivi-1 and its derivatives for combination therapies with platinum drugs in the treatment of drug resistant tumors.

## MATERIALS AND METHODS

### Cell culture

The human breast carcinoma cell line MDA-MB-231, non-small cell lung carcinoma H1299 were obtained from American Type Culture Collection (ATCC). LN-428 glioblastoma cells were kindly provided by Dr. Robert W. Sobol (University of Pittsburgh Cancer Institute). Cal33 head and neck cancer cells were kindly provided by Dr. Jennifer R. Grandis (University of Pittsburgh Cancer Institute). 983A melanoma cells were kindly provided by Dr. Stergios J. Moschos (University of North Carolina). Cisplatin sensitive ovarian cancer cells A2780 and their cisplatin resistant derivative cells A2780cis were obtained from Sigma-Aldrich (St. Louis, MO). Bax/Bak WT and double knockout MEF cells were established by Dr. Stanley J. Korsmeyer, and kindly provided by Dr. Shivendra Singh (University of Pittsburgh Cancer Institute). Drp1 WT and knockout MEF cells were established by Dr. Katsuyoshi Mihara, and kindly provided by Dr. Kasturi Mitra (University of Alabama). Cells were cultured in either RPMI 1640 or DMEM media supplemented with 10% heat-inactivated fetal calf serum and 1% penicillin-streptomycin in 5% CO_2_ at 37°C. Ovarian cancer patient ascites were obtained under an IRB protocol, IRB0406147, approved by the University of Pittsburgh Cancer Institute. Primary epithelial ovarian cancer cells (EOC) presented in those ascites were isolated and cultured as described previously [36].

### Plasmid, siRNA and transfection

pDsRed2-Mito plasmid was obtained from Clontech (Palo Alto, CA), and Noxa specific siRNA was obtained from Dharmacon (Lafayette, CO). DNA transfection was performed using FuGENE 6 (Roche Diagnostics, Indianapolis, IN) and siRNA transfection was performed using oligofectamine (Invitrogen, Carlsbad, CA) according to the manufacture's instructions.

### Reagents

Mdivi-1 and its analogs were obtained from Sigma-Aldrich and Key Organics Ltd (Camelford, Cornwall, UK). Other reagents, unless specified, were from Sigma-Aldrich.

### Cell proliferation and cytotoxicity assay

Cell proliferation was determined using a CyQUANT Direct Cell Proliferation Assay kit (Invitrogen), the activity of caspase-3/7 was measured using a Caspase-Glo 3/7 Assay Systems (Promega, Madison, WI), and the MTS colorimetric survival assay was performed using the CellTiter 96 AQueous One Solution Cell Proliferation Assay kit (Promega, Madison, WI), according to the manufacturer's instructions. The survival fractions were calculated after setting untreated control cells at 100%. The data were plotted and curve fitted using GraphPad Prism software. The cytotoxic interactions between the two drugs was determined using the method of Chou and Talalay [20]. The combination index (CI) was determined using a computer program CompuSyn. A CI < 1 indicates synergy, CI > 1 indicates antagonism, and CI = 1 indicates additivity. To quantify the number of cells with active caspase-3, cells were fixed with 4% paraformaldehyde (Electron microscopy sciences, Hatfield, PA), stained with Alexa Fluor-488 conjugated antibody against cleaved caspase-3 (Asp175) (Cell Signaling Technology, Danvers, MA) followed by flow cytometry. An FITC Annexin V Apoptosis Detection Kit (BD PharMingen, San Diego, CA) was used to quantify apoptotic cells, according to the manufacturer's instructions.

### Cell cycle analysis

Cells were treated with 20 μM cisplatin, 20 μM mdivi-1, or the combination of 20 μM cisplatin and 20 μM mdivi-1 for 20 h. S phase cells were then pulse-labeled with 10 μM BrdU for 30 min at 37°C. Cells were trypsinized, fixed in 70% ice-cold ethanol, and incubated overnight at 4°C. DNA was denatured in 2 N HCl containing 0.5% Triton X-100, and neutralized with 0.1 M Na_2_B_4_O_7_. Cells were then stained with FITC-labeled anti-BrdU antibody (BD Biosciences, San Jose, CA). To determine the number of cells in mitosis, cells were then stained with Alexa Fluor 647-conjugated phospho-Histone H3 (Ser 10) antibody (Cell signaling technology). To measure DNA content, cells were incubated in PI solution (PBS containing 50 μg/ml of PI and 40 μg/ml of RNase A) for 30 min at room temperature. Samples were analyzed on a CyAn ADP Analyzer (Beckman Coulter, Brea, CA). Data were analyzed using Summit software.

### ATP measurement

Total cellular ATP content was determined using a luminescent ATP detection kit, ATPlite (PerkinElmer Life Sciences, Boston, MA), according to the manufacturer's instruction. The luminescence intensity was measured using a microplate reader, Synergy 2 (BioTek instruments, Winooski, VT).

### Extracellular flux (XF) analysis

Oxygen consumption rate (OCR) and extracellular acidification rate (ECAR) were measured using a Seahorse XF24 Extracellular Flux Analyzer (Seahorse Bioscience, North Billerica, MA), as previously described [37]. Cells were seeded in XF24 cell culture plates at 4×10^4^ cells/well and incubated in 5% CO_2_ at 37°C. Prior to the analysis, cells were washed and growth medium was replaced with bicarbonate-free medium. Cells were then incubated for another 60 min in a 37°C incubator without CO_2_, followed by simultaneous OCR and ECAR measurements.

### Mitochondrial membrane potential and ROS generation

To measure mitochondrial membrane potential and intracellular generation of ROS, cells were incubated with 50 nM of TMRM (Invitrogen) and 10 μM DCF-DA (Sigma) for 20 min at 37°C after drug exposure. To measure mitochondrial generated ROS, cells were pre-incubated with 5 μM of MitoSox (Invitrogen) for 30 min at 37°C then followed by drug exposure. After wash with PBS, cells were trypsinized and suspended in HBSS containing 1% BSA. The fluorescence intensity of TMRM, DCF, and MitoSox were analyzed using a CyAn ADP Analyzer (Beckman Coulter, Brea, CA) or an Accuri C6 flow cytometer (BD Accuri Cytometers, Ann Arbor, MI).

### Western blot analysis

Whole cell lysate was prepared by lysing cells in cell lysis buffer (Cell signaling technology) containing complete protease inhibitor (Roche). Cell lysates were then cleared at 15,000 rpm for 15 min at 4°C. To determine the extent of cytochrome c release, cells were permeabilized using 50 μg/ml digitonin in PBS containing complete protease inhibitor on ice for 10 min, and then the cytosolic fraction and heavy membrane fraction were separated by centrifugation at 4,000 × g for 5 min at 4°C. The protein content was quantified using a Bio-Rad Protein Assay kit (Bio-Rad Laboratories, Hercules, CA). The equal amount of protein was separated on Tris-glycine gels (Invitrogen). The separated proteins were blotted onto a polyvinylidene difluoride membrane and blocked overnight at 4°C in phosphate-buffered saline containing 0.1% Tween 20 and 10% nonfat dry milk (blocking buffer). Membranes were incubated with primary antibodies in blocking buffer overnight at 4°C. Primary antibodies used were: Drp1 and Cytochrome c from BD Biosciences; β-actin and ATM from Sigma; Chk1, phospho-Chk2 (Thr68), Chk2, Mcl-1, Bax, Bak, Bid, Puma, Bik, Bcl-xL, Caspase-9 and cleaved Caspase-3 from Cell Signaling Technology; phospho-Chk1 (Ser317) from R&D systems; phospho-ATM (S1981) from Epitomics; phospho-Histone H2AX (Ser 139) and Noxa from Millipore; Complex IV subunit I from MitoSciences. Membranes were then washed and incubated in peroxidase conjugated anti-rabbit IgG (Sigma) or anti-mouse IgG (Sigma) secondary antibody for 1 h at room temperature. Membranes were developed using SuperSignal West Femto Maximum Sensitivity Substrate (Thermo Fisher Scientific).

### Immunofluorescence

Cells grown on cover slides were fixed with 4% paraformaldehyde in PBS for 15 min at 37°C. After wash with PBS, cells were mounted with VECTASHIELD mounting medium containing DAPI (Vector Laboratories, Burlingame, CA). Confocal images were captured using a laser-scanning confocal microscope, Olympus FLUOVIEW FV-1000, with a PlanApo N 60x oil immersion objective, NA=1.42 (Olympus).

### Electron microscopy

Cells cultured in 35 mm culture dishes were fixed with 2.5% glutaraldehyde in PBS for 1 hour at room temperature, and post-fixed for 1 hour at 4°C in 1% OsO_4_ with 1% K_3_Fe(CN)_6_. After dehydration and embedding, ultrathin (70 nm) sections were cut and mounted onto copper grids. Sections were stained with 2% uranyl acetate followed by 1% lead citrate, and imaged using a JEOL JEM 1011 transmission electron microscope (Peabody, MA) at 80 kV. Digital images were taken on an AMT 2K digital camera (AMT, Danvers, MA).

## SUPPLEMENTARY FIGURES


