# Supplementary material for: Novel combination of mitochondrial division inhibitor 1 (mdivi-1) and platinum agents produces synergistic pro-apoptotic effect in drug resistant tumor cells
Source: Oncotarget. 2014 May 4;5(12):4180–94. doi: 10.18632/oncotarget.1944 (PMC4147315; doi:10.18632/oncotarget.1944)
Supplement: Supplementary file 1 [file oncotarget-05-4180-s001.pdf]

Novel combination of mitochondrial division inhibitor 1 (mdivi-1) and platinum agents produces synergistic pro-apoptotic effect in drug resistant tumor cells

Supplementary Material

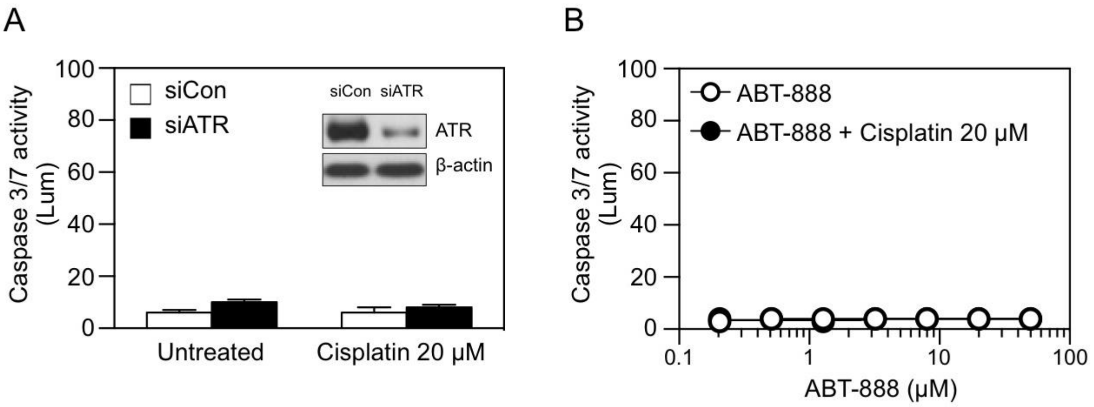

Supplemental Figure 1: MDA-MB-231 cells are resistant to ATR and PARP inhibition alone and in combination with cisplatin (A) MDA-MB-231 cells were transfected with control or ATR siRNA for four days, and then left untreated or treated with cisplatin for 20 h. (B) MDA-MB-231 cells were treated with series doses of PARP inhibitor ABT-888 alone or with the combination of cisplatin for 20 h. Apoptosis was determined by measuring the activity of caspase-3/7. These data represent the mean ± s.d.; $n = 4$.

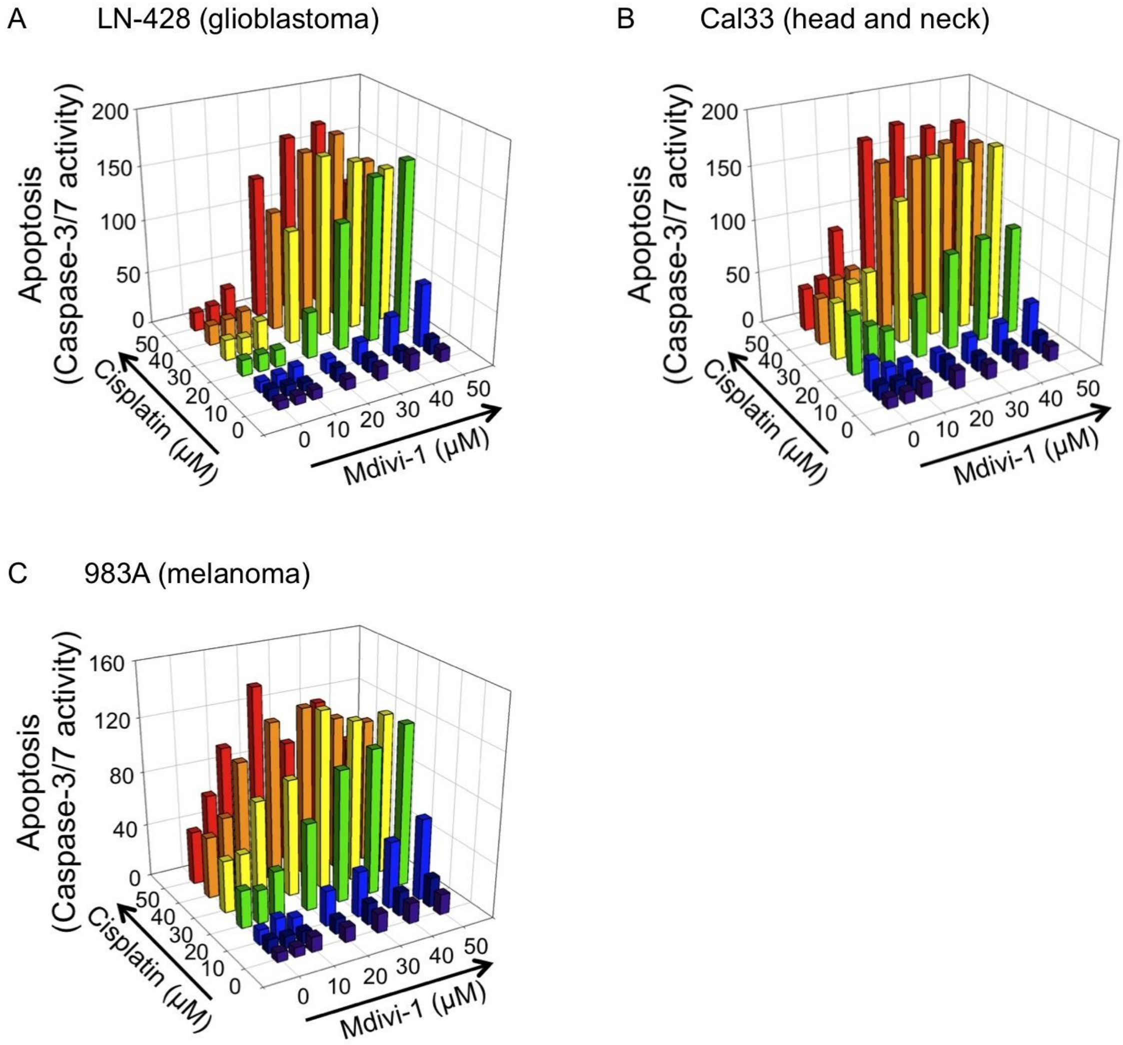

Supplemental Figure 2: Enhanced apoptosis by the combination of cisplatin and mdivi-1 in various types of cancer cells.Human glioblastoma (LN-428), head and neck cancer (Cal33), and melanoma (983A) cells were treated with various combinations of cisplatin and mdivi-1 at indicated concentrations. After 20 h, apoptotic cell death was determined by measuring the activity of caspase-3/7.

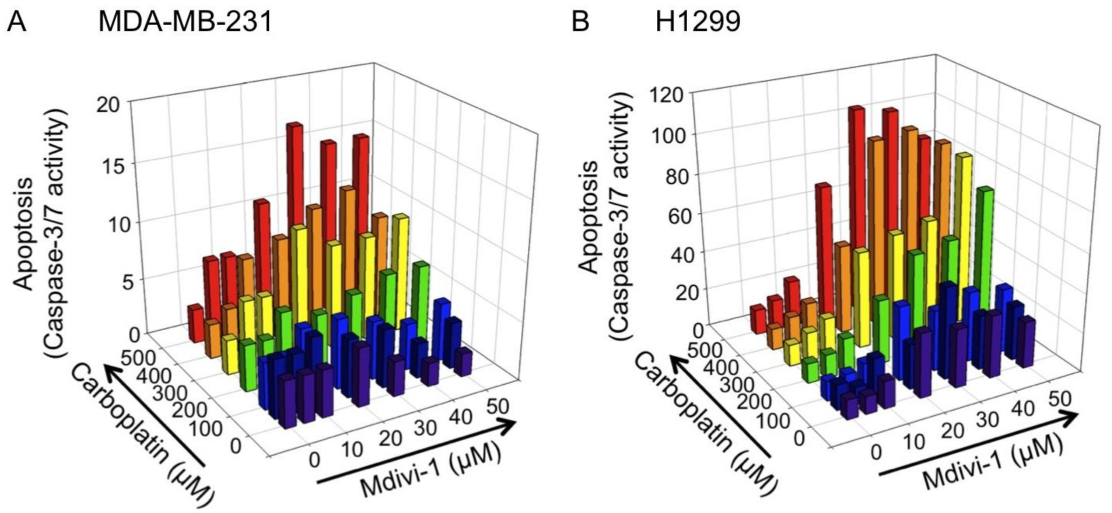

Supplemental Figure 3: The synergistic effect between mdivi-1 and carboplatin.Human breast carcinoma MDA-MB-231 and non-small cell lung carcinoma H1299 cells were treated with various combinations of carboplatin and mdivi-1 at indicated concentrations. After 20 h, apoptotic cell death was determined by measuring the activity of caspase-3/7.

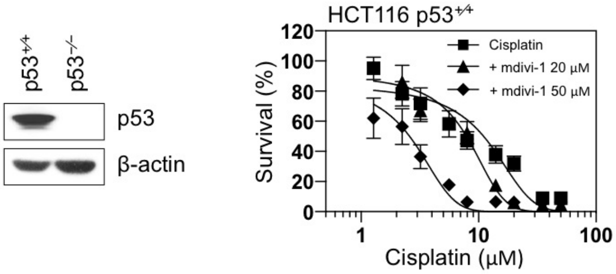

Supplemental Figure 4: The enhanced cell death by the combination of cisplatin and mdivi-1 is p53 independent p53 wild-type (p53 +/+) and knockout (p53 -/-) HCT116 colon cancer cells were treated with increasing doses of cisplatin alone or with the presence of 20 µM or 50 µM of mdivi-1 for 72 h. Cell survival was determined by MTS assay. These data represent the mean ± s.d.; *n* = 4.
